# 

**Published:** 1853-05

**Authors:** 


### CONTENTS.

| | PAGE. |
|---|---|
| Albuminate of Iron and Soda, | 328 |
| Aphonia Relived by Iodine Inhalations, | 350 |
| American Association, Transactions of, | 363 |
| Asthma, Spasmodic, | 365 |
| Ascarides, | 15 |
| Artificial Urethra, Puncture of the Bladder, | 42 |
| American Medical Association, | 70 |
| Asphyxia, Pathology of, | 188 |
| American Medical Association, | 70, 240, 511 |
| American Association for the Advancement of Science, | 280 |
| Aneurism, Thoracic, | 426 |
| Asphyxia Infantile, Remedy for, | 57 |
| Anderson, a few Thoughts by, | 236 |
| Action of Medicines in the System | 561 |
| Alabama, Medical Association of, | 213 |
| | |
| Biliary Calculi, Obliteration of the Biliary Duct and Abscess of the Liver, | 7 |
| Biliary Calculi, Disease of Heart, | 81 |
| Blindness, Color, | 120 |
| Bomard's Experiments, | 185 |
| Beaumont Dr., Death of, | 272 |
| Belladonna in Vomiting in Pregnancy, | 468 |
| Blood, Venous and Arterial, Difference in Temperature in, | 483 |
| Brainard, Professor, Departure of, | 512 |
| Books Received, | 355, 512 |
| Bismuth, Sub. Mur., Action of, | 197 |
| Botany, Principles of, | 209 |
| | |
| Constipation with Vomiting, | 49 |
| Chemical Examination of the Urine, | 58, 113 |

Catalogue of Medical Plants, U. S., . . . . 67
Clinical Phrase Book, . . . . . 69
Chloride of Sodium, Influences of on Absorption and Exhalation, 524
Cook Co. Medical Society, . . . 79, 217, 276
Cases, Reports of, . . . . . 84
Cold Water in Dysentery, . . . . 152
Charleston Med. Jour., Editorial Correspondence of, . . 177
Conception, its Relation to Menstruation and Lactation, . 185
Cramp or Spasm, . . . . . 291
Castration with Thumb Nail, . . . . 234
Chronic Abscess of Breast, Treatment of, . . . 260
Chlorate of Potash in Ulcerative Stomatitis, . . . 269
Children, Diseases of, . . . . . 497
Maternal Management of, . . . 509
Chloride of Soda, . . . . . 270
Collodion for Chordee, . . . . . 270
Chloroform, Deaths by, . . . . . . 271, 465
Croup, Membranous, . . . . . 296
Cholera Infantum, Quinine in, . . . . 323
Credit to Whom Credit is Due, . . . . 367
Chloroform in Obstruction of the Bowels from Spasm, . . 415
City Mortality of New Orleans, . . . . 417
Chloroform, Inhalation of in Pneumonia, . . . 472
Correspondence of Boston Medical Journal, . . . 330
Cinch. Ferrata, Tr. of, . . . . . 335
Chloroform, Impending Death from, . . . . 485

Diet, Remedial Agent, . . . . . 51
Druggist Receipt Book, . . . . . 69
Digitaline, . . . . . . 91
Dyspepsia, Nit. Silver in, . . . . . 122
Diabetes, Effects of Different Remedies in, . . . 265
Daguerreotype Art, Influence of the Practice of, . . 278
Dysentery, Saline Treatment of, . . . . 366
Delirium Relieved by Chloroform, . . . . 414
Davis on Pathology of Fever, . 17, 97, 161, 241, 305, 385, 432, 529

Ergot in Retention of Urine, . . . . 94
European Correspondence, . . . . . 513
Epilepsy, Physiology of, . . . . . 145
Epilepsy, Influence of Posture in, . . . . 196
Erectile Tumor of the Orbit, . . . . 545
Exostosis of the Condyle, Etc., . . . . 553

Experiments, &c., Post Mortem, . . . 324
Eloquent Extract, . . . . . 422
Epilepsy of two Years' Standing, . . . 431
Elastic Cups, . . . . . . 482

Fracture Box for Leg, . . . . 90
Fever, Pathology of, . . 17, 97, 161, 241, 305, 385, 432, 529
Fish of the West, . . . . 244
Fœtus Killed by Lightning, . . . . 269
Fly Poison, . . . . . . 271
Fracture Tables, . . . . . 353
Fever, Yellow, in New Orleans, . . . . 511

Humulus Lupulus, Anti Periodic Properties of, . . 44
Horner, Professor, Death of, . . . . 47
Hygiene, Female, . . . . 68
Homeopath, . . . . . . 206
Homeopathic Practice, Success of, . . . 239
Hyperæsthesis Cerebralis, . . . . 516
Histology, Pathological, Atlas of, . . . 288
Hallucination, Singular, . . . . 381
Hospital, King's College, . . . . 476
Hydrophobia, . . . . . . 482
Hernia, Cases of, . . . . . 486
Hospital Reports, . . . 298, 369, 417

Insanity, Cases of, . . . . . 1
Intussusception, . . . . . 297
Iowa Medical Journal, . . . . 362
Illinois State Medical Society, . . . 131
" " " " Transactions of, . . 506

Lardner's Medical Philosophy, . . . . 70
Lycoperdon Proteus, Anæsthetic Properties of, . 285
Lactine as an Article of Nutrition, . . 267
Lasalle Co. Medical Society, . . . . 275
Lead as a Deodorizer, . . . . . 481

Miasm in Inflammation of Lungs, . . . 38
Menstruation, Absence of in a Mother, . . . 45
Medical Men, Duty of, . . . . . 121
Medical Teaching in Paris, . . . . 125
Montreal Journal of Medicine, . . . . 212

Milk Sickness, . . . . . 225
Minnesota Medical Convention, . . . 363
Medical Societies, . . . . . 425
Microscopist, . . . . . . 503
Medical Reporter, . . . . . 363

Neuralgia, Oxalate of lime, relation to, . . 157
Neuralgic Operations, . . . . 257
Nephretis, Desquamative, . . . . 347
N. W. Medical Society, . . . . 142

Ovariotomy, case of, . . . . . 489
Obliteration of Lachrymal Sac, . . . 423
Obstetric practice, . . . . . 9
Occulsion of the Vagina, . . . . 551
Osteo-Aneurism, . . . . . 118
Opiate poisoning, tracheotomy for, . . . 205
Opium administered by urethra, . . . 268

Paris, Hospitals of, . . . . . 430
Plumbi Acetas, internal use of, . . . 408
Physic, great capacity of, . . . . 368
Pneumonia, by Smellfungus, . . . . 37
Phlebetis-portal, . . . . . 41
Pneumonia, . . . . . 123
Pneumonia Intermittent, . . . . 495
Pneumo Thorax, Post Mortem in, . . . 155
Ptyalism, hot water and soup in, . . . 263
Pereira, death of, . . . . . 287
Placenta, developement of in fallopian tube, . . 290
Parasites, influence of, in disease, . . . 333
Paracentesis thoraces, . . . . 336
Peninsular Journal of Medicine, . . . 362

Respiration Subservient to Nutrition, . . . 212
Rush Med. College, . . . . 367, 512
Rheumatism Treated by Quinine, . . . 413

Syphilis, Iodide of Sodum in, . . . . 36
Saccharimeter, Soleils, . . . . 68
Stricture, Treatment of, . . . . 225
Small Pox and Vaccination, . . . . 527
Surgical Cases, . . . . . 490

Table Tipping, Physiology of, . . . . 401

Tape Worm, Remedy for, . . . . . . 6

Turpentine, Uses of, . . . . 556

Tetanus, Chloroform in, . . . . . 264

" Successfully Treated, . . . . 321

Uva Ursi, Substitute for Ergot, . . . . 467
Ulcers, Treatment of, . . . . . 272
" " " by Quinine, . . . . 429

Vaccination, . . . . . . 182

What to Observe, . . . . . 207
Wisconsin Medical Society, . . . . 273
" " Association, . . . . 274
Williams, Dr. Stephen W., . . . . . 428
Wilde on Diseases of the Ear, . . . . 567
Walton's Operative Ophthalmic Surgery, . . . 566

### RECEIPTS

### FOR THE JOURNAL DURING THE MONTH OF APRIL.

### Illinois.

| | |
|---|---|
| Dr. Jas. P. Dinniwitt, | $2 00 |
| " John Garrison, | 2 00 |
| " Wm. F. Coleman, | 2 00 |
| " O. P. Hathaway | 2 00 |
| " W. T. Briscoe, | 2 00 |
| " G. Smith, | 2 00 |
| " Hosea Davis, | 1 00 |
| " A. W. Butler, | 2 00 |
| " Roberts, | 2 00 |
| " J. B. Nash, | 2 00 |
| " N. C. Hunt, | 2 00 |
| " G. H. Young, | 2 00 |
| " Geo. Ryen, | 2 00 |
| " S. Wiley, | 2 00 |

### Michigan.

| | |
|---|---|
| Dr. F. Myers, | 2 00 |

### Wisconsin.

| | |
|---|---|
| Dr. J. L. Ingersoll, | 2 00 |
| " Cutting Marsh, | 2 00 |

### Indiana.

| | |
|---|---|
| Dr. J. Gregory, | 2 00 |
| " M. Bill, | 2 00 |

### Iowa.

| | |
|---|---|
| Dr. Wm. Rinehart, | 4 00 |
| " Francis Brady, | 2 00 |

